# PI3K/mTOR inhibition promotes the regression of experimental vascular malformations driven by *PIK3CA*-activating mutations

**DOI:** 10.1038/s41419-017-0064-x

**Published:** 2018-01-19

**Authors:** Laura di Blasio, Alberto Puliafito, Paolo Armando Gagliardi, Valentina Comunanza, Desiana Somale, Giulia Chiaverina, Federico Bussolino, Luca Primo

**Affiliations:** 1 Candiolo Cancer Institute FPO-IRCCS, 10060 Candiolo, Torino Italy; 2 Department of Oncology, University of Torino, 10100 Torino, Italy

## Abstract

Somatic activating mutations within the *PIK3CA* gene have been recently detected in sporadic lymphatic and venous malformations, and in vascular malformations (VM) associated to overgrowth syndromes, such as CLOVES and Klippel–Trenaunay syndrome. Although VM are often limited to specific tissue areas and can be well treated, in extended or recurrent lesions novel therapeutic approaches are needed. We generated a mouse model of VM by local expression of *PIK3CA-*activating mutation in endothelial cells. *PIK3CA-*driven lesions are characterized by large areas of hemorrhage, hyperplastic vessels, infiltrates of inflammatory cells, and elevated endothelial cell density. Such vascular lesions are ameliorated by administration of dual PI3K/mTOR inhibitor, BEZ235, and mTOR inhibitor, Everolimus. Unexpectedly, the expression of *PIK3CA*-activating mutations in human endothelial cells results in both increased proliferation rates and senescence. Moreover, active forms of *PIK3CA* strongly promote the angiogenic sprouting. Treatment with PI3K/mTOR inhibitors restores normal endothelial cell proliferation rate and reduces the amount of senescent cells, whereas treatment with Akt inhibitor is less effective. Our findings reveal that *PIK3CA* mutations have a key role in the pathogenesis of VM and *PIK3CA*-driven experimental lesions can be effectively treated by PI3K/mTOR inhibitors.

## Introduction

Somatic mutations within the phosphatidylinositol-4,5-bisphosphate 3-kinase catalytic subunit alpha (*PIK3CA)* gene, coding for the p110α catalytic subunit of class 1A phosphoinositide-3-kinase (PI3K), are frequently found in human tumors^1^. Most of these are activating mutations, clustering in two hot spot regions in helical (E542 and E545) and kinase domains (H1047) of the PI3K protein^2^.

Recently, numerous studies reported the presence of somatic *PIK3CA*-activating mutations, including those traditionally defined as “cancer-associated”, in several genetic syndromes characterized by overgrowth^3^. The identification of *PIK3CA* somatic mutations in syndromes with distinct, but partially overlapping, clinical findings, such as Fibroadipose hyperplasia or Overgrowth^4^, CLOVES syndrome^5^, macrodactyly and muscle hemihypertrophy^6^, Megalencephaly-Capillary Malformation^7^, and hemimegalencephaly^8^, suggested to group all of these syndromes and term them “PIK3CA-related overgrowth spectrum” (PROS)^3^.

Most PROS syndromes are characterized by vascular malformations (VMs), suggesting that *PIK3CA* somatic mutations could occur in vascular endothelial cells (EC). Indeed, *PIK3CA-*activating mutations have been detected in lymphatic EC isolated from tissues of PROS syndromes^9^, and in lymphatic and venous malformations which arise as an isolated VM^10–13^. Furthermore, earlier studies on mouse models showed that alteration of PI3K signaling resulted in severe vascular defects. For example, mice deficient in the p110α catalytic subunit of PI3K, display multiple vascular defects, including dilated vessels in the head and reduced branching morphogenesis in the endocardium^14^. Interestingly, only p110α activity, but not p110β and p110γ, is essential for vascular development^15^. Similarly, mice deficient in expression of PI3K regulatory subunits in ECs display a vascular phenotype during development as well as during tumor neovascularization^16^.

Accumulating studies suggest a critical role for mutations in *PIK3CA* gene as driver event in vascular diseases. Cellular and mouse models expressing *PIK3CA*-activating mutations are characterized by serious phenotypic alterations, strongly supporting the causative effect of these mutations in the pathogenesis of PROS syndromes and VMs^11–13,17,18^.

However, biological mechanisms involved in the pathogenesis of VMs, both sporadic and associated to other defects, are poorly described.

To overcome these limitations we combined the design of genetically modified mouse models with conditional endothelium-specific expression of *PIK3CA*-activating mutations and the use of primary endothelial cultures. Our mouse models allow to mimic a localized disease by ectopic activation of the mutation and to verify the role of such mutations in vascular development. We show that expression of *PIK3CA*-activating mutations in human ECs results in both increased proliferation rates and senescence. We report evidence of treatment efficacy of PI3K/mTOR inhibitors both *in vitro* and *in vivo* by reverting morphology and functionality of altered ECs and vasculature.

## Results

### Endothelial expression of *Pik3ca*^*H1047R*^ mutation is embryonically lethal

We investigated the effects of PIK3CA-activating mutations on vascular development *in vivo* by crossing *Pik3ca*^*H1047R*^ mice to the *Tie2Cre* mouse strain, in which *Cre* expression is restricted to endothelial compartment. This promoter is not completely specific for ECs but has the advantage of being expressed during early development^19^. Cre-mediated deletion of loxP-flanked transcriptional stop cassette allows for tissue-specific expression of the mutant allele.

**Fig. 1:**
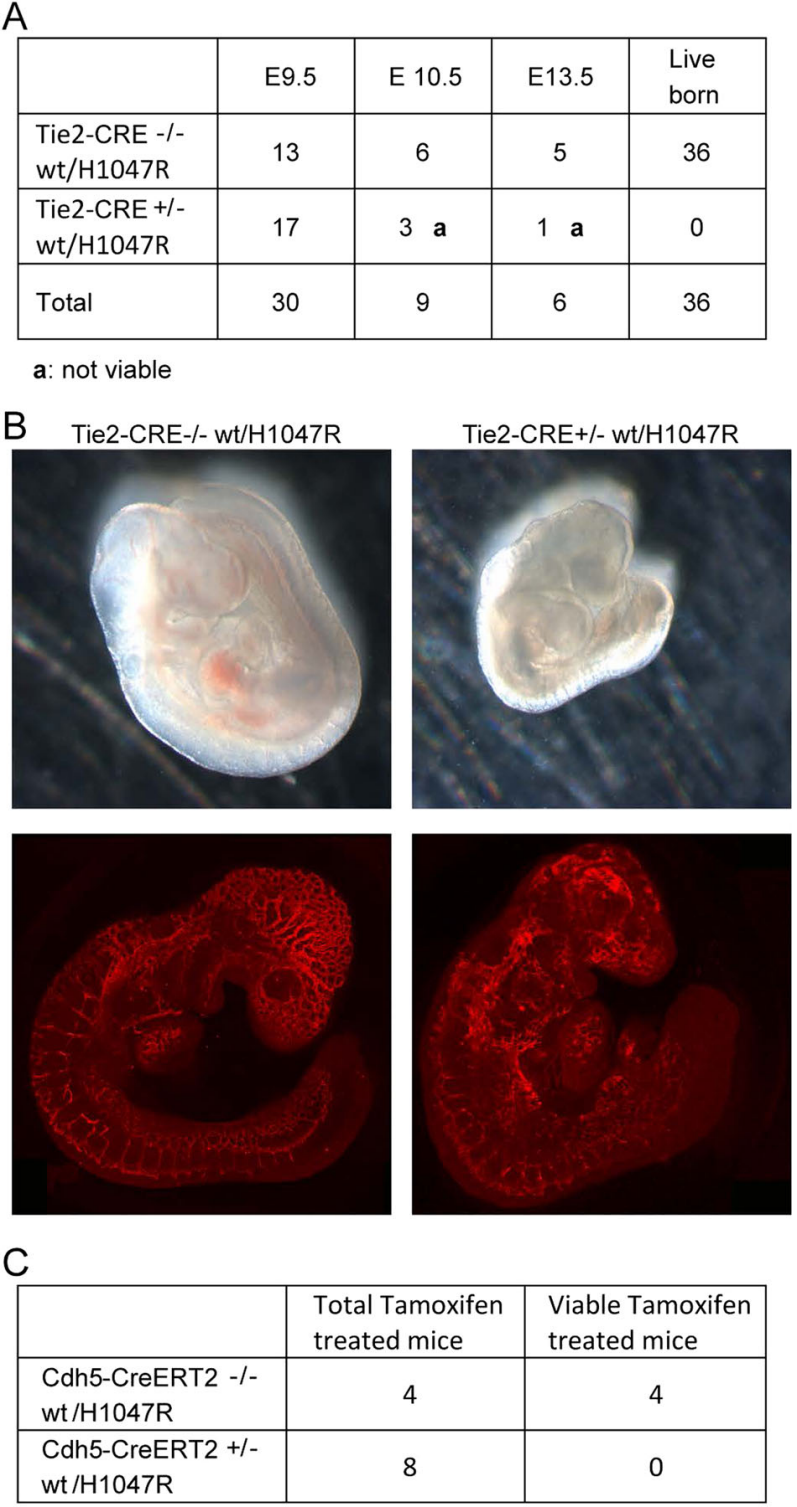
Mice expressing *Pik3ca*^*H1047R*^ in developing and adult vascular EC are not viable **a** Transgenic mice that express latent *Pik3ca*^*H1047R*^ mutant allele (H1047R) were crossed with mice expressing *Cre* recombinase under endothelial promoter (*Tie2Cre*). The Mendelian expected ratio for endothelial *Pik3ca*^*H1047R*^ allele was the 50% of newborn mice, but only mice carrying wild-type alleles were identified. **b** We recovered live embryos with PIK3CA mutations until mouse embryonic day 9.5. These embryos showed growth delay (top) and evident vascular defects (bottom, in red endomucin staining). **c** Transgenic mice that express latent *Pik3ca*^*H1047R*^ mutant allele (H1047R) were crossed with mice expressing Tamoxifen-inducible *Cre* recombinase under VE-Cadherin promoter (*Cdh5-CreERT2*). Mice treated with single administration of Tamoxifen did not survive >2 weeks after *Cre* induction

No *Tie2Cre:Pik3ca*^*H1047R*^ pups were born and longitudinal analysis of embryos revealed that lethality was occurring prior to E10.5 (Fig. 1a). At E9.5, mutant embryos were smaller and developmentally delayed compared to wild-type litter-mates (Fig. 1b). Although E9.5 *Tie2Cre:Pik3ca*^*H1047R*^ mutant embryos were observed to have a heartbeat, they showed a disorganized and truncated vascular network (Fig. 1b). Whole-mount staining for ECs revealed that mutant embryos had formed the major vessel branches of the dorsal aorta and anterior cardinal veins but had failed to undergo vessel remodeling and sprouting in the head, somites and dorsal regions of the embryo (Fig. 1b).

The lethal phenotype is consistent with the fact that genetic evidence of heritable syndromes with activating mutation in *PIK3CA* gene has never been reported. In contrast, postzygotic mutations have recently been described in PROS syndromes^5^. With this motivation in mind we evaluated the effects of *Pik3ca*^*H1047R*^ mutation in the adult vasculature. By crossing *Pik3ca*^*H1047R*^ mice to mice expressing a Tamoxifen-inducible recombinase *Cre* under control of VE-cadherin promoter (*Cdh5-CreERT2)* we were able to obtain conditional expression of *PIK3CA*-activating mutation in vascular ECs upon administration of Tamoxifen. We treated mice with a single intraperitoneal administration of Tamoxifen resulting in 100% of mortality in *Pik3ca*^*H1047R*^/ *Cdh5-CreERT2* mice at 15 days after injection (Fig. 1c). In contrast *Pik3ca*^*wt*^/ *Cdh5-CreERT2* mice did not show any sign of suffering and appeared completely normal. To understand the cause of death of *Pik3ca*^*H1047R*^/ *Cdh5-CreERT2* mice, we sacrificed three mice 13 days after Tamoxifen administration and we analyzed multiple organs. We observed signs of a cardiac degenerative process, with small spots of fibrosis (Fig. S1A, circled areas) and vacuolated cardiomyocytes (Fig. S1A’, arrows). Conversely, the other organs analyzed (brain, liver, kidneys, spleen, lungs) did not show any defects.

### *PIK3CA*-activating mutations induce both cell senescence and proliferation in ECs

**Fig. 2:**
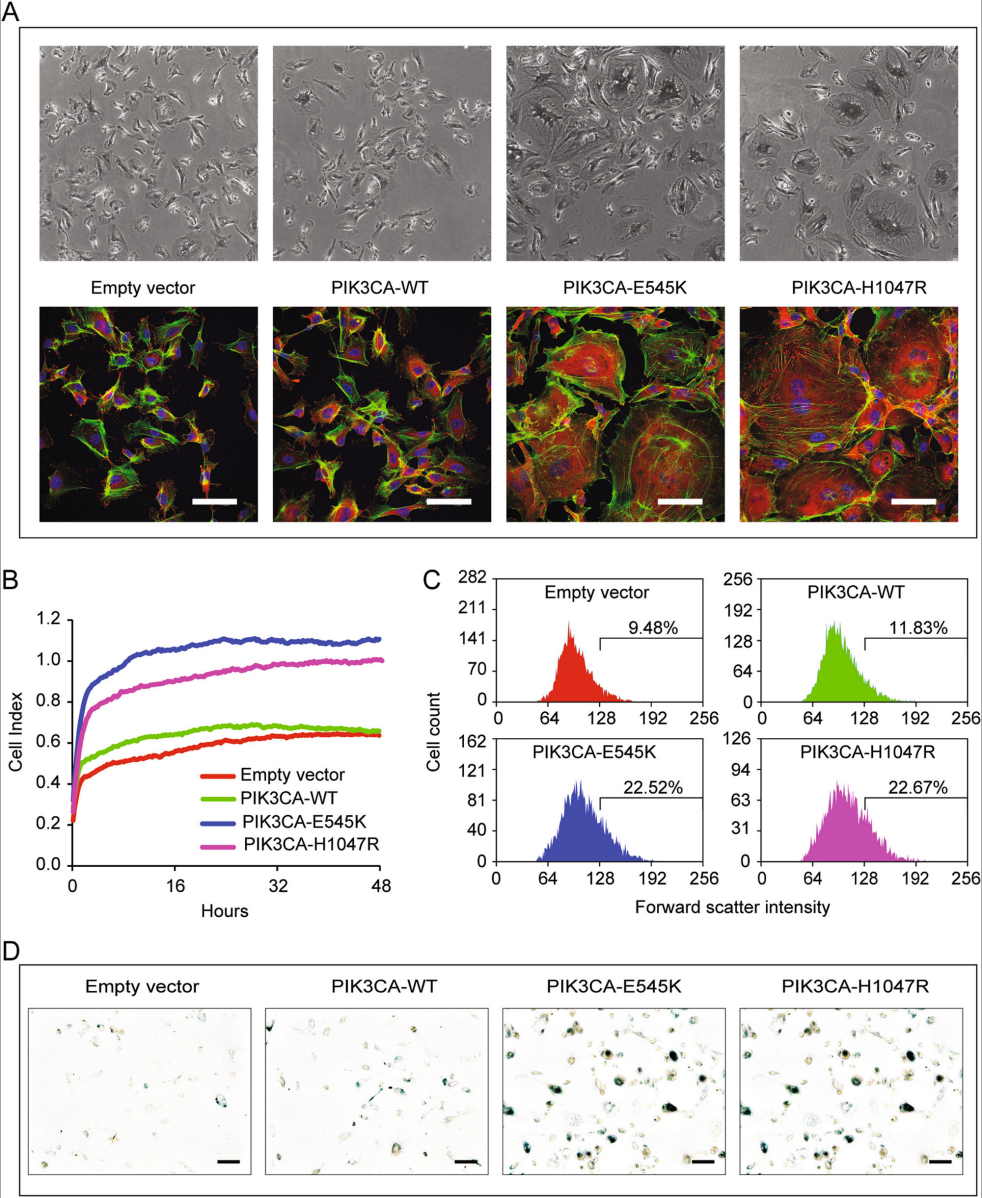
PIK3CA-activating mutations induce morphological alterations and senescence in EC **a** Human primary endothelial cells were transduced with retroviral vectors carrying activating mutations of PIK3CA (PIK3CA-H1047R and PIK3CA-E545K), wild-type PIK3CA (WT), or with empty vector. EC-expressing PIK3CA mutants showed morphological alterations. Large cells with abnormal stress fibers and intracellular vesicles are observable (green: Phalloidin, red: vinculin, blue: DAPI). Scale bar 50 µm. **b** Cell surface area is measured in real-time by impedance system with xCELLigence technology in absence of growth factors. EC-H1047R and EC-E545K adhere on substrate and occupy more surface than EC-WT or normal cells. Data were plotted as the mean cell index from three wells at each time points; *P*-values were calculated at 24 h, *P < 0.005, vs. control wild-type PIK3CA endothelial cells. **c** Transduced EC are analyzed by flow cytometer for forward scatter intensity. The expression of PIK3CA mutants increase the percentage of cells with higher volume as indicated. The reported experiments are representative of three independent experiments. **d** β-galactosidase staining show senescent cells. Image manipulation has been done to highlight positive cells, as described in the Methods section. Original pictures are in supplemental Fig. 1c

To investigate in detail the effects of *PIK3CA*-activating mutations in the endothelium, we expressed H1047R mutant in human umbilical vein endothelial cells (HUVEC) by retroviral infection. We also generated cells expressing *PIK3CA-E545K*, a different activating mutation detected in VM syndromes. As control cells, we used HUVECs infected with an empty retroviral vector alone or one expressing wild-type human *PIK3CA* (Fig. S1B). The expression of active PI3K evidently modified EC morphology by dramatically increasing average cell size. Among those expressing *PIK3CA-H1047R* and *PIK3CA-E545K*, we observed several large cells, while the expression of wild-type *PIK3CA* did not induce any obvious morphological abnormality (Fig. 2a). We measured the adhered cell surface and we observed a twofold increase of total area in EC-H1047R and EC-E545K compared with *PIK3CA-WT* or empty vector expressing EC (Fig. 2b) plated at the same cell density. Interestingly, the larger surface occupied by EC-expressing *PIK3CA* mutants is mainly caused by the increased size of a small amount of cells, as shown by flow-cytometric analysis (Fig. 2c). These cells showed several phenotypic aspects normally associated to senescence, such as the presence of a large number of internal vesicles, the increased cell size and, frequently, the presence of multiple nuclei. Therefore we evaluated the expression of β-galactosidase, a recognized senescence marker, on *PIK3CA*-mutant EC^20^. Indeed the expression of active PI3K, both H1047R and E545K mutants, increased the amount of β-galactosidase positive cells (Fig. 2d and S1C–D).

**Fig. 3:**
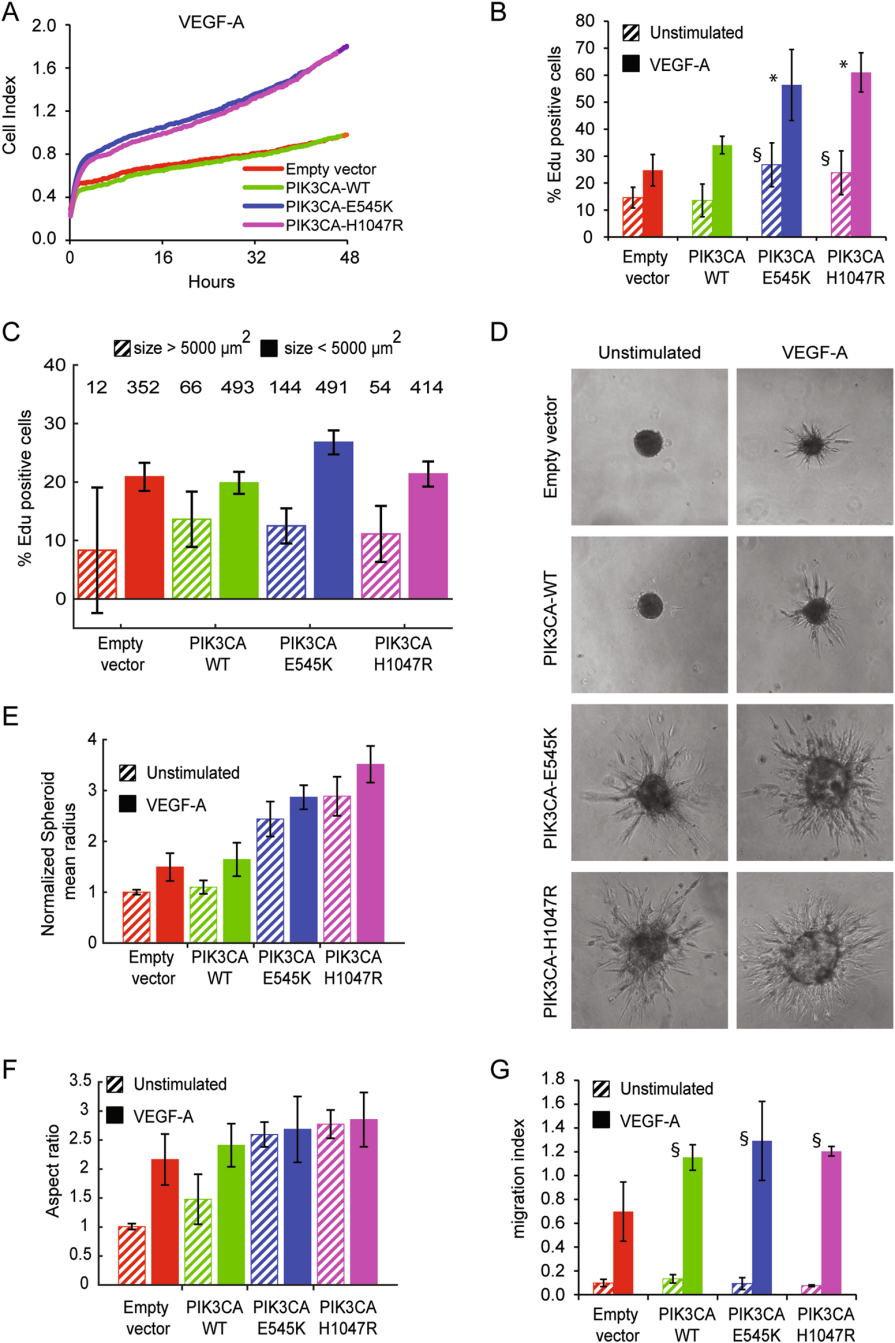
PIK3CA-activating mutations increase proliferation rate and sprouting formation **a** Cell surface area is measured in real-time by impedance system with xCELLigence technology in presence of VEGF-A. EC- H1047R and EC-E545K grew faster than EC-WT or normal cells. Slopes for each sample are reported in supplemental Fig. 1e. **b** DNA replication rate was quantified by Click-iT EdU Alexa Fluor 647 Imaging kit. EC-H1047R and EC-E545K showed higher DNA replication rates. The percentage of EdU-positive nuclei is indicated in the graph. Data were plotted as the mean from three independent experiments; *P < 0.005 and ^§^*P* < 0.001, vs. control wild-type PIK3CA endothelial cells. **c** After Click-iT EdU assay, VEGF-A-stimulated EdU-positive cells are divided into two categories: normal ( < 5000 µm^2^) and large (>5000 µm^2^); the percentage of positive large cells among the total number of large cells, and correspondingly the percentage of positive normal cells among the total number of normal cells was calculated. For each category, total number of cells in the category is indicated on the top of the graph area. **d** Spheroids of the indicated EC were embedded in a collagen gel and stimulated or not with VEGF-A to generate capillary-like sprouts. Spheroids of EC-expressing active forms of PIK3CA produced sprouts even in absence of VEGF-A. **e** Quantification of spheroids growth. Equivalent radii of the spheroids were normalized with control average radius (unstimulated empty vector EC). **f** To quantify the sprouting, an aspect ratio measure was used, defined as the ratio between the equivalent ratios obtained by perimeter and area of the spheroids. **g** Chemotaxis assay was performed by means of Boyden Chamber; EC were induced to migrate by VEGF-A. Data were plotted as the mean from three independent experiments; ^§^*P* < 0.001, vs. endothelial cells transduced with empty vector

Cell senescence is commonly characterized by low replicative rates, so we expected to find reduced proliferation rates in EC-expressing active PI3K. Instead, in presence of vascular endothelial growth factor (VEGF)-A, overall, cells were found to proliferate more rapidly than normal EC or EC-expressing *PIK3CA-WT* (Fig. 3a and S1E). To decouple the potential effect of increased cell surface from that of increased proliferation rates in these conditions, we measured DNA replication rates by means of EdU incorporation assay. EC-H1047R and EC-E545K showed higher DNA replication rates, which were particularly elevated when EC were stimulated with VEGF-A (Fig. 3b). The increase of DNA replication even in absence of VEGF-A is apparently in contrast with results presented in Fig. 2b that shows lack of area change in the different cell lines overtime. However, the time variation of the total area covered by cells is due to both proliferation and the balance between normal and senescent cells in the population. Interestingly the coexistence of highly proliferating and senescent cells is consistent with non-neoplastic lesions observed in human syndromes. To support this idea, we analyzed the cell size of EdU-positive EC stimulated with VEGF-A (Fig. 3c). To elucidate whether large putatively senescent cells are also non-proliferating, we calculated the percentage of large cells (area >5000µm^2^) that were positive for EdU, and we obtained that only (11.1±4.8)% of EC-H1047R and (12.5±3.0)% of EC-E545K were proliferating among the large cells while the corresponding percentages among normal cells were (21.4±2.2)% and (26.7±2.1)%, respectively. Therefore, the likelihood of being proliferating among large cells is roughly twice as small as for normal cells, thus reinforcing the notion that putative senescent cells are for the majority non-proliferating (Fig. 3c).

### ECs expressing PIK3CA-activating mutations sprout forming capillary-like structures

To assess the possible relevance of morphological and proliferative alterations induced by *PIK3CA* mutations in physiological endothelial processes, we used an angiogenesis sprouting assay. In this assay EC spheroids, embedded in a collagen gel, generate multicellular protrusions, reminiscent of sprouting capillaries. Normal EC spheroids, as well as those expressing *PIK3CA-WT*, formed sprouts only when stimulated by VEGF-A (Fig. 3d–f). Surprisingly, spheroids of EC-expressing active forms of PI3K produced many sprouts even in absence of angiogenic stimuli, while the addition of VEGF-A induced a larger number of capillary-like structures than in control EC (Fig. 3d–f). Excessive sprouting angiogenesis could be either caused by greater ability of EC to migrate or by increased proliferation alone. To investigate the role of EC migration, we performed a chemotaxis assay where EC are induced to migrate directionally by a spatial gradient of VEGF-A (Fig. 3g). Both wild-type PI3K and active mutants increased motility of EC in presence of VEGF-A but, according to our experiments, EC-H1047R and EC- E545K did not show significant difference in migratory ability compared to cells expressing *PIK3CA-WT* (Fig. 3g). This result suggested a prominent role for cell proliferation in increased angiogenic sprouting caused by *PIK3CA*-activating mutants.

### Localized expression of *Pik3ca*^H1047R^ in mice induces VMs

**Fig. 4:**
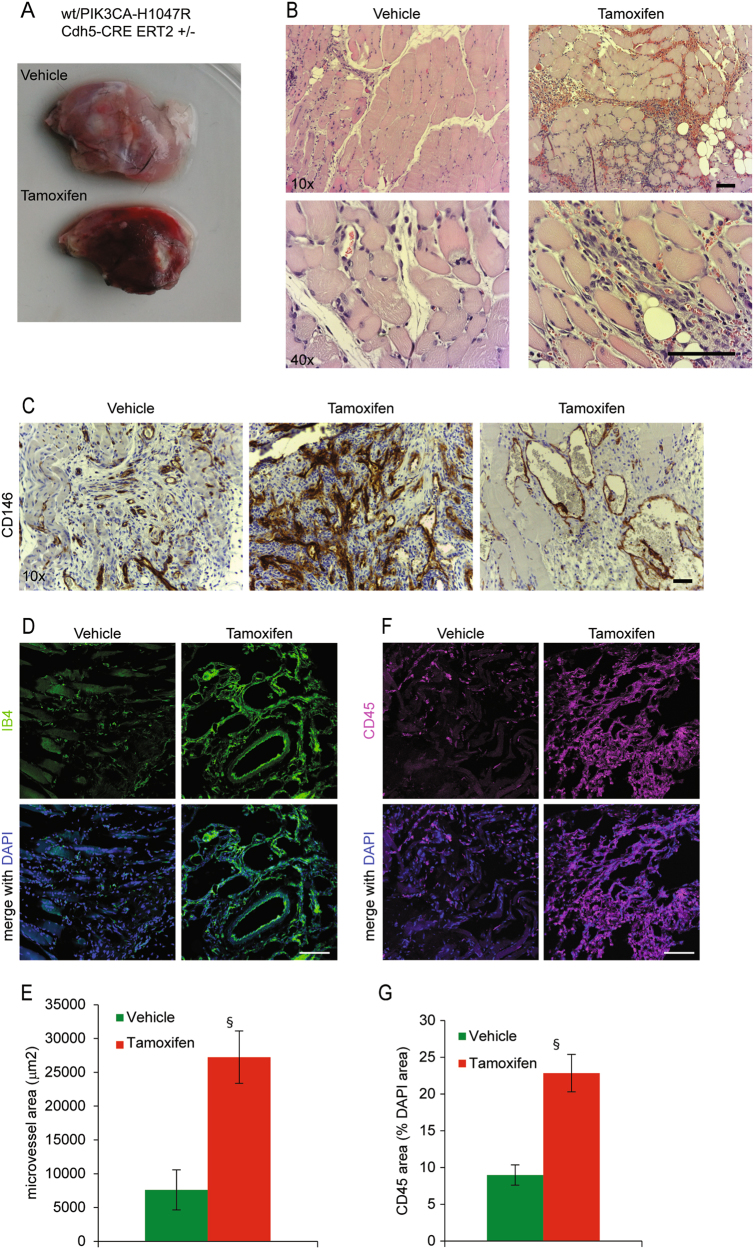
Localized expression of *Pik3ca*^H1047R^ in mice induces vascular malformations **a**, **b** Vehicle or 4-OH Tamoxifen were locally injected in posterior limbs of *Pik3ca*^H1047R^/*Cdh5-CreERT2* mice. After 1 week, animals were sacrificed and muscles were dissected and analyzed by H&E staining. **c** Vessels of the same samples were analyzed by immunohistochemistry with anti-CD146 antibody. **d**, **f** Frozen tissues of vehicle or 4-OH Tamoxifen-injected mice were analyzed by immunofluorescence with anti-IB4 to stain vessels (green in **d**, scale bar 100 µm) and anti-CD45 to stain recruited inflammatory cells (magenta in **f**, scale bar 100 µm). **e**, **g** Quantification of microvessel area (IB4-positive area in the total field area) and CD45-positive area in samples from vehicle or 4-OH Tamoxifen-injected mice; ^§^*P* < 0.005, vs. vehicle treated

The lethal phenotype obtained with the expression of *Pik3ca*^H1047R^ in whole vascular endothelium both during development and in adult mice, prompted us to conceive a model in which the expression was spatially confined. For this reason, we intramuscularly injected posterior legs of *Pik3ca*^H1047R^/ *Cdh5-CreERT2* mice with 4-OH Tamoxifen. A single administration was sufficient to induce the appearance of evident signs of bleeding and vessel abnormalities after 1 week (Fig. 4a). Hystopathological analysis revealed enlarged vessels and increased vessel density with angiomatous lesions, and large areas of infiltrated inflammatory cells (Fig. 4b). In pathological tissues, vessels are formed by a thick heterogeneous layer of EC, frequently disorganized and discontinuous, with the presence of clusters of cells heterogeneous in size (Fig. 4c–e). Large vessels appeared dilated, occasionally forming hemangioma-like structures (Fig. 4c–e). Moreover, pathological lesions showed recruitment of inflammatory cells potentially caused by production of chemoattractant molecules (Fig. 4f, g). Notably, the secretion of cytokines is one of the recognized signs of senescence, so-called secretory associated senescence phenotype^21^. In fact, tissues from 4-OH Tamoxifen-treated mice showed positivity for β-galactosidase staining (Fig. S1F) and increased β-galactosidase activity (Fig. S1G). Moreover, the senescence marker p15^INK4B^ clearly accumulated in pathological tissue (Fig. S1H).

### The dual PI3K/mTOR inhibitor BEZ235 completely rescues the cell phenotype induced by PIK3CA*-*activating mutations

**Fig. 5:**
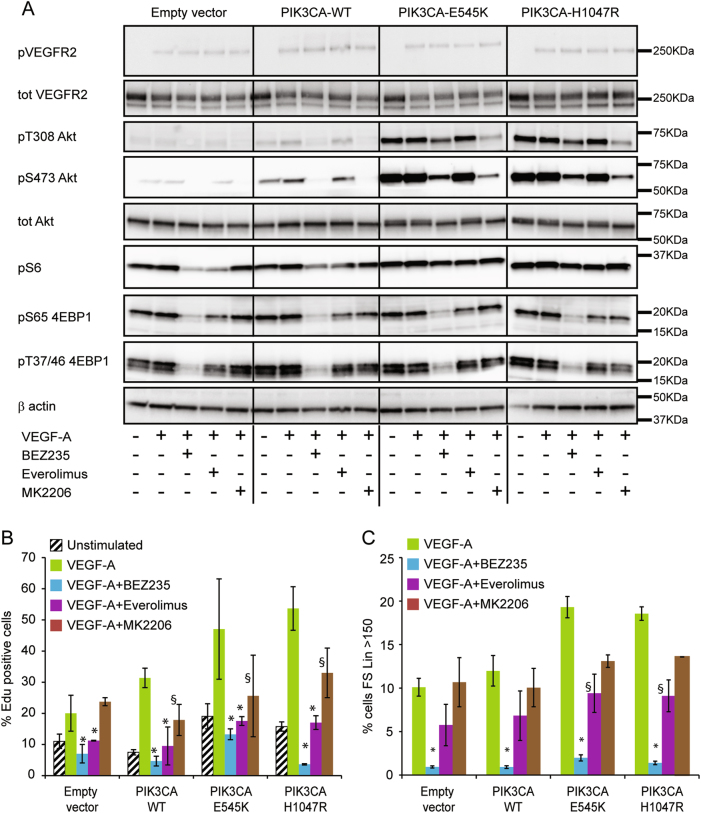
The dual PI3K/mTOR inhibitor BEZ235 rescues the cell phenotype induced by PIK3CA*-*activating mutations **a** EC were serum starved and then stimulated with VEGF-A in presence or absence of the indicated inhibitors (BEZ235, Everolimus, MK2206); corresponding lysates were then separated by SDS-PAGE and analyzed with the indicated antibodies. **b** DNA replication rate was measured by Click-iT EdU Alexa Fluor 647 Imaging kit after treatment of EC with the indicated inhibitors. The percentage of EdU-positive nuclei is indicated in the graph. Data were plotted as the mean from three independent experiments; *P < 0.001 and ^§^*P* < 0.05, vs. the same VEGF-stimulated and untreated cells. **c** EC treated with the indicated inhibitors were analyzed by flow cytometer. The percentage of cells with linear Forward Scatter > 150 (large cells) is indicated in the graph. Data were plotted as the mean from three independent experiments; *P < 0.01 and ^§^*P* < 0.05, vs. the same VEGF-stimulated and untreated cells

Taking advantage of the availability of several inhibitors of PI3K/mTOR signaling pathway developed as cancer drugs, we specifically evaluated the effect of Everolimus (mTOR inhibitor) MK2206 (Akt inhibitor) and BEZ235 (double mTOR/PI3K inhibitor) on our models. Treatments with MK2206 and BEZ235 were able to blunt Akt phosphorylation in parental and PI3K WT-expressing EC, whereas in EC-H1047R and EC-E545K Akt phosphorylation level was only partially reduced (Fig. 5a and S2A). EC treated with Everolimus or BEZ235 showed also a decreased phosphorylation of S6, a substrate of mTOR (Fig. 5a and S2B). However, in EC-H1047R and EC-E545K, phospho-S6 was not reduced by treatment with these inhibitors. Conversely, BEZ235 was able to reduce the phosphorylation of another substrate of mTOR, 4EBP1, both on serine 65 and threonines 37 and 46, also in EC-H1047R and EC-E545K (Fig. 5a and S2B). Similar signal alterations were also observed in absence of VEGF-A stimulation (Fig. S2C-E).

Despite the limited efficacy of these drugs in inhibiting the signaling pathway activated by mutated PIK3CA, the biological effects on EC were impressive. Treatment with BEZ235 rescued the phenotype induced by PIK3CA-H1047R or PIK3CA-E545K expression, completely inhibiting cell proliferation in VEGF-A-stimulated conditions (Fig. 5b). Similar results were obtained with Everolimus, which completely abrogates the proliferation increase induced by *PIK3CA*-activating mutations. In contrast, Akt inhibition only partially reduced the proliferation of EC-expressing both *PIK3CA-WT* and *PIK3CA* mutants and was less effective, compared with the other two inhibitors, to reduce the proliferation rate of VEGF-A stimulated normal EC (Fig. 5b). Interestingly, BEZ235-treated cells recovered normal cellular size just after 72 h of treatment with a clear reduction of the number of large/senescent cells (Fig. 5c and S3A–C). Time-lapse microscopy experiments showed that in presence of BEZ235, senescent cells rapidly died while non-senescent cells stopped to proliferate (Supplementary movies 1–4). Treatment with BEZ235 and Everolimus was also able to reduce the angiogenic sprouting from spheroids of EC stimulated with VEGF-A (Fig. S3D-E).

### Vascular *PIK3CA*-driven lesions are ameliorated by BEZ235 administration

**Fig. 6:**
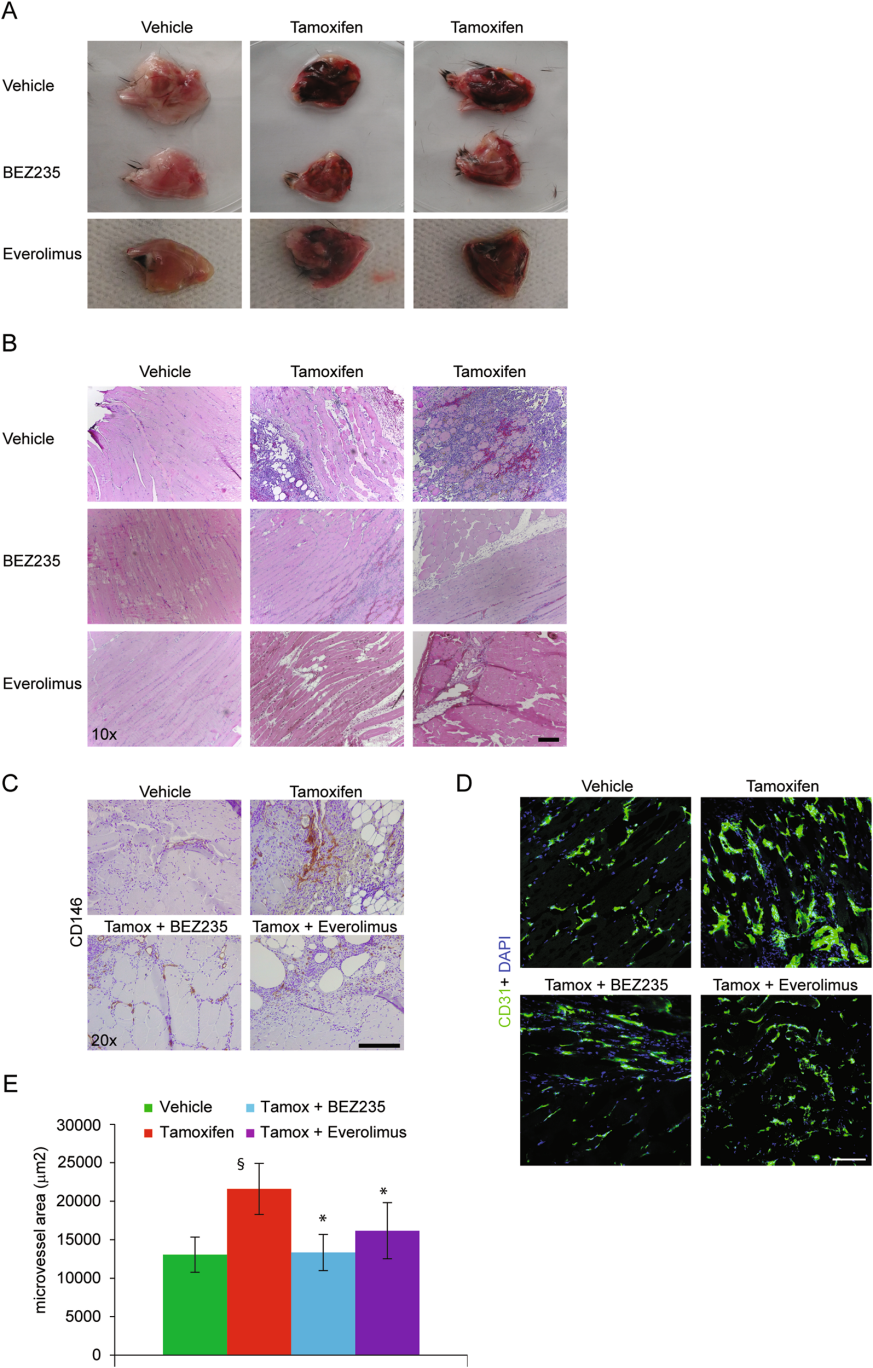
Vascular PIK3CA-driven lesions are ameliorated by BEZ235 administration **a**, **b** Localized *Pik3ca*^*H1047R*^ expression was induced in mice by 4-OH Tamoxifen injection in the posterior leg. One week later, we started the treatment with BEZ235 or Everolimus. Explanted muscles were examined and sections of the same tissues were analyzed by H&E. **c** Vessels of the same samples were analyzed by immunohistochemistry with anti-CD146 antibody. **d** Frozen tissues of vehicle or 4-OH Tamoxifen-injected mice, treated or not with BEZ235 or Everolimus, were analyzed by immunofluorescence with anti-CD31 to stain vessels (in green, merged with DAPI in blue). **e** Microvessel area (CD31-positive area in the total field area) was quantified; ^§^*P* < 0.05, vs. vehicle treated, *P < 0.05 vs. Tamoxifen treated

The impressive results obtained by treating EC-expressing *PIK3CA* mutations with BEZ235 or Everolimus prompted us to evaluate its effect on *in vivo* vascular lesions. Localized *Pik3ca*^*H1047R*^ expression was induced in mice by 4-OH Tamoxifen injection in the posterior leg. One week later we started the treatment with BEZ235 or Everolimus. Examination of explanted leg muscles indicated that both treatments reduced vascularization and bleeding (Fig. 6a). Analysis of histological sections confirmed this evaluation showing normalization of tissue treated with BEZ235, although large infiltrates of immune cells were still present (Fig. 6b). The effects of Everolimus were less pronounced, with histological sections still characterized by extensive areas of pathologic tissue and bleeding (Fig. 6b). Notably, both treatments did not simply block the progression of vascular lesions but reduced the entity of the pathological traits, and re-established a normally vascularized tissue (Fig. 6c), as it was also demonstrated by the quantification of CD31-positive microvessel density (Fig. 6d, e).

## Discussion

Vascular anomalies, including both vascular tumors and malformations represent a broad spectrum of disorders ranging from a simple “birthmark” to life-threatening entities^22^. These diseases frequently affect infants and children but symptoms can also become apparent in young adults. VMs are often limited to specific tissue areas and can be treated in severe cases by surgical resection or sclerotherapy. However, in extended or recurrent lesions, alternative therapeutic approaches are needed. Unfortunately, no drugs have been proven to be effective so far.

Germline and somatic activating mutations in the gene encoding the EC tyrosine kinase receptor Tie2 have been reported to be causative for familial and for some sporadic venous malformations, respectively^23,24^. More recently, the discovery of somatic activating mutations of *PIK3CA* gene in venous and lymphatic malformations has opened new perspectives for therapeutic options^9–13^.

Here we showed that expression of *PIK3CA-*activating mutations in human EC induced hyperproliferation and concomitant cellular senescence. *PIK3CA*-induced senescent cells are larger than normal EC and are characterized by many intracellular vesicles and elevated β-galactosidase activity. Oncogene-induced senescence was described >10 years ago for the *Ras* oncogene in human fibroblasts^25^, whereas there is no clear evidence for a role of PI3K signaling in cell senescence^26^. However, recent interesting studies showed that treatment of senescent cells with the mTOR inhibitor abrogates the senescent-associated secretory phenotype, whereas cell cycle arrest remains unaffected^27,28^. Our observations indicate that a constitutive active PI3K signaling is able to promote the emergence of non-proliferating, senescent cells in absence of global reduction of endothelial proliferation. We can speculate that concomitant EC senescence and proliferation might limit VMs to hyperplastic forms, such as enlarged vessels or clusters of EC, without progressing towards more aggressive neoplastic forms. Notably, treatment with Everolimus, which block mTOR1, completely restores the normal proliferation rate in EC-expressing PIK3CA mutations but appears less effective in hampering cell senescence. Instead, the PI3K/mTOR inhibitor BEZ235 eliminates all senescent cells, rapidly inducing their apoptosis.

How can this phenotype of mixed proliferating/senescent cell affect the functionality of EC? The most striking result is the increased *in vitro* 3D sprouting induced by VEGF-A. Even more impressive is that mutant EC were able to form new sprouts even in the absence of VEGF-A. The role of PIK3CA in angiogenesis both during development and tumor progression is well established^29^. However, traditionally, VMs are lesions composed only of mature vessels, although microvascular proliferation in up to 30% of congenital VMs has been reported, especially in those with a significant arteriovenous component^30^. High density of small EC clusters, which are bona fide new capillaries, is evident also in our animal model. Our model is generated by local expression in mouse limb of *Pik3ca*^H1047R^ in VE-cadherin-positive EC, and is characterized by large areas of hemorrhage, hyperplastic vessels, infiltrates of inflammatory cells and, precisely, elevated EC density. These pathological properties mirror several clinical signs observed in patients with VMs, although the diversity of diseases classified as VMs makes it difficult to associate our model to a specific syndrome^31^. Moreover, VE-cadherin is commonly expressed in all vessels, including veins, arteries, capillaries and lymphatics, thus our model do not precisely phenocopy venous, lymphatic or capillary malformations but represents a mixed vascular disease.

It is interesting that PIK3CA-activating mutations have been detected in PROS syndromes, which are frequently characterized by VM such as CLOVES and Klippel–Trenaunay syndromes^9^, but in which other cellular types are affected as well. What is the relation between PIK3CA-activating mutations in EC and the pathogenesis of PROS is not known.

One hypothesis is that mutations arise in progenitors cells able to differentiate to several cell types, including EC, and that the severity of the disease could depend on the period of emergence of mutation. Indeed, our mice model of constitutive expression in EC of PIK3CA^H1047R^ in Tie2-positive and VE-cadherin-positive cells together indicates that broad and early expression of PIK3CA^H1047R^ during development is not compatible with life. These results are in agreement with recent published results and are consistent with the lack of reported hereditary diseases driven by mutations in the *PIK3CA* gene^17^.

Particularly interesting are the results obtained by treating mice with BEZ235 or Everolimus. Indeed, BEZ235 is particularly effective to reduce the lesions induced by active PIK3CA, and allows an almost complete recovering of the normal phenotype with only residual infiltrate of inflammatory cells. Similarly, mice treated with Everolimus showed signs of disease regression although hemorrhage and vascular alterations were still present. These observations are extremely relevant from a translational point of view. Although clinical trials with Everolimus-analogs in patients with VMs and PROS syndromes are ongoing, to our best knowledge no clinical study is currently evaluating PI3K-inhibitors or PI3K/mTOR inhibitors, such as BEZ235. Further preclinical experiments with these drugs on models of PIK3CA-driven diseases would prompt new clinical trials, which could change the management of these syndromes.

## Materials and methods

### Mice strains and treatment

R26-*Pik3ca*^H1047R^ strain was purchased from Jackson Laboratory. These mice carry loxP-flanked transcriptional stop cassette upstream of the H1047R mutant allele of *Pik3ca* within the Gt(ROSA)26Sor locus^32^. The *Tie2Cre* transgenic mice (Jackson Laboratory) have the mouse endothelial-specific tyrosine kinase receptor Tie2 promoter directing the expression of *Cre* recombinase^19^. R26-*Pik3ca*^H1047R^ and *Tie2Cre* transgenic mice were crossed to obtain the endothelial expression of H1047R mutant allele during embryogenesis. The progeny was analyzed postnatally and at different developmental stages (E9.5, 10.5, and 13.5) by genotyping and whole-mount immunofluorescence of the embryos. *Cdh5-CreERT2* mice (kindly provided by professor R. Adams) have the Tamoxifen-inducible *Cre* recombinase (*CreERT2*) under the regulation of the vascular endothelial cadherin promoter (Cdh5)^33^. R26-*Pik3ca*^H1047R^ and *Cdh5-CreERT2* mice were crossed to induce the endothelial expression of H1047R mutant allele during adulthood. After genotyping, 8–10 weeks of age mice carrying both the transgenes and control mice were intraperitoneal injected with 1 mg Tamoxifen (Sigma, dissolved in ethanol/peanut oil [10:90 v/v]) for either 5 consecutive days or only 1 day to induce systemic endothelial expression of mutant PIK3CA protein. After 13 days, mice were killed and different tissues (heart, brain, liver, kidneys, spleen, lungs) were dissected and analyzed by H&E staining. Alternatively, posterior left limbs of 8–10 weeks of age mice carrying both the transgenes and control mice were injected once with 1 mg 4-OH Tamoxifen (Sigma, 10 μg/μl in ethanol) to induce local endothelial expression of mutant PIK3CA protein into the muscle. After 15 days, the latter mice were sacrificed and the tissue was analyzed by immunohistochemistry. When used, BEZ235 and Everolimus were administered to mice by oral gavage as follow: BEZ235 at the dose of 25 mg/kg in NMP (*N*-Methyl-2-pyrrolidone)/PEG300 (Poly Ethylene Glycol 300) (10/90 v/v), Everolimus at the dose of 5 mg/kg in phosphate-buffered saline (PBS), both for 7 consecutive days, 1 week after 4-OH Tamoxifen injection. All the genotype analysis was performed by PCR amplification following providers’ instructions. All animals were maintained in the animal facility of the Candiolo Cancer Institute under germ-free housing conditions. All animal experiments were performed in compliance with guidelines governing the care of laboratory mice and approved by Italian Ministry of Health.

### Whole-mount embryo immunofluorescence

Freshly isolated embryos were fixed overnight in 4% paraformaldehyde at 4° C, washed in PBT (PBS, 0.1% Tween 20) and subjected to dehydration in increasing methanol concentration (50%, 80% methanol/PBT, 100% methanol) followed by rehydration in decreasing methanol concentration (80%, 50% methanol/PBT, PBT). After washing in Pblec (PBS pH 6.8, 1% Tween 20, 1 mM CaCl_2_, 1 mM MgCl_2_, 0.1 mM MnCl_2_), the embryos were incubated overnight at 4° C in the presence of rat anti-mouse endomucin (Santa Cruz Biotecnologies) diluted 1:20 in Pblec. After five washes in PBT, embryos were incubated overnight at 4° C with goat anti-rat Alexa 555 (ThermoFisher Scientific) diluted 1:100 in PBS, 0.5% BSA and 0.25% Tween 20, followed by washing in PBT and post-fixation in 4% paraformaldehyde before analysis.

### Immunohistochemistry analysis

Paraffin samples of mice limbs from each group of treatment were serially cut (10 μm) and rehydrated through 100% xylene and 100, 95, and 70% ethanol before immersion in H_2_O. Sections were then stained with hematoxylin and eosin (H&E) and dehydrated. Rabbit anti-CD146 antibody (Abcam) to bind vessels and rabbit anti- p15^INK4B^ antibody (ThermoFisher Scientific) to visualize senescent cells were used, after heat-induced epitope retrieval in sodium citrate buffer (1 mM sodium citrate, pH 6.6), inactivation of endogenous peroxidases in 3% H_2_O_2_ and blocking in TBS/0.1% Tween/0.3% Triton/5% goat serum. HRP-conjugated goat anti-rabbit secondary antibody and DAB (3,3′-Diaminobenzidine) substrate were used to stain vessels.

### Cell culture

Human EC were isolated from umbilical cord veins, characterized and grown in M199 (Sigma) containing 20% fetal bovine serum (FBS, ThermoFisher Scientific), bovine brain extract, heparin (50 μg/ml, Sigma) and penicillin–streptomycin (200 U/ml, Sigma) on 1% gelatin-coated tissue culture dishes, as previously described^34^. Gryphon amphotropic packaging cells were grown in IMDM (Sigma) supplemented with 10% FBS, l-Glutamine (2 mM, Sigma) and antibiotics. β-galactosidase staining was performed with the Cell Signaling Technology Senescence staining kit following manufacturer instructions. To enhance contrast of β-galactosidase staining we removed background and enhanced contrast in each channel (R, G, B). The same values of relative contrast were used on all images. Original pictures are reported in fig S1C and S3B. Quantification of the staining was done by manually counting positive and negative cells in at least six images for each condition from two independent experiments. Furthermore, β-galactosidase activity was also evaluated on either cell- or tissue-lysates using Mammalian β-galactosidase Assay Kit (ThermoFisher Scientific) following manufacturer instructions, with the exception of incubation time that was extended to 24 h.

### Retroviral vector construct and EC infection

The cDNAs of wild-type and mutant PIK3CA (E545K and H1047R) inserted into the pBabe retroviral vector were purchased from Addgene. The amphotropic cell line Gryphon was transfected with retroviral vectors, and the retroviral supernatants obtained were collected, filtered and supplemented with 4 μg/ml of polybrene (Sigma-Aldrich). Medium of EC were replaced with the appropriate retroviral supernatants, and cells were incubated at 37° C with 5% CO_2_ for 6 h. 72 h after infection, successfully infected cells were selected with Puromycin (2.5 μg/ml, Sigma) and analyzed for PIK3CA protein expression by western blot.

### Cell size and cell proliferation analysis

Cell size and cell proliferation were evaluated with xCELLigence system (Roche), as previously described^35^. In brief, 96-well microtiter plates that are specifically designed to measure cellular impedance (E-Plate, Roche) were coated with 1% gelatin for 1 h at 37° C and then saturated with 3% bovine serum albumin. In total, 2 × 10^3^ EC were resuspended in 0.1 ml of medium supplemented with 10% FBS with or without VEGF-A (20 ng/ml, R&D System) and transferred on E-Plates after background measurement. The extent of cell proliferation and spreading, measured as changes in impedance, was monitored every 15 min for a total period of 48 h. The measured impedance was expressed as relative impedance (Cell Index). The Cell Index at each time point is defined as (Rn-Rb)/(15 Ω), where Rn is the cell-electrode impedance of the well when it contains cells and Rb is the background impedance of the well with the media alone.

Cell proliferation was also analyzed by Click-iT EdU Alexa Fluor 647 Imaging kit. In brief, 10 × 10^3^ EC were plated on gelatin-coated glass coverslips in 24-well plates in 0.5 ml of medium supplemented with 10% FBS with or without VEGF-A (20 ng/ml) for 24 h. When used, the following inhibitors were added at the concentration of 100 nM: BEZ235, Everolimus and MK2206 (all from Selleckchem). EdU was added to cells and left for another 24 h. Then cells were fixed and stained following manufacturer instructions. Four random fields of each sample from three independent experiments were photographed at the confocal microscope at low magnification and Alexa Fluor 647 positive nuclei were counted. To measure the proportion of EdU-positive cells and their size we performed the same experiment on a glass bottom multiwell plate followed by a counterstaining with DAPI and VE-Cadherin (R&D Systems). For each experiment (performed in duplicate) a large (2.2 mm by 1.6 mm) image was acquired using a Biotek Cytation reader (×10 objective). The VE-Cadherin staining channel was used to extract cell outlines by means of Ilastik^36^ and overlapping cells were manually separated in the images. Cell outlines were then used to measure cell size. EdU and DAPI staining channels were also segmented and cells with overlapping DAPI and EdU staining were considered positive. Segmentation and statistical analysis were performed by means of custom written MATLAB algorithms. The fraction of putative senescent cells was identified as that of cells with area larger than 5000 µm^2^. This threshold was chosen according to the area distribution of the different mutants and by manually picking putative senescent cells. To calculate the fraction of EdU-positive cells in each category (<5000 µm^2^, normal and >5000 µm^2^, large) we just counted the number of positive large cells among the total number of large cells, and correspondingly the number of positive normal cells among the total number of normal cells. The error was calculated assuming that those numbers were distributed according to a Poisson statistics. The final error was calculated with standard error propagation methods.

### Immunofluorescence microscopy

To analyze cell morphology, EC were plated on gelatin-coated glass coverslips in 24-well plates. After 3 h of adhesion, cells were fixed with 4% paraformaldehyde in PBS for 10 min. After fixation, cells were rinsed three times with PBS, quenched with 50 mM NH_4_Cl for 20 min at room temperature, washed twice with PBS, and then permeabilized with PBS 0.2% Triton X-100 for 8 min at room temperature. After two washes with PBS, coverslips were blocked with PBS 1% donkey serum for 1 h at room temperature, and incubated with anti-paxillin (BD Biosciences) primary antibody overnight at 4° C in a humidified chamber. After three washes with PBS, coverslips were incubated for 1 h at 37° C in a humidified chamber with fluorescent secondary antibodies (donkey anti-rabbit Alexa Fluor 555, ThermoFisher Scientific) and Phalloidin-Alexa Fluor 488 (ThermoFisher Scientific). Coverslips were then rinsed three times with PBS, mounted, and analyzed using a confocal laser-scanning microscope (TCS SP2 with DM IRE2; Leica) equipped with ×63/1.40 HCX Plan-Apochromat oil-immersion objective. Confocal images are the maximum projections of a *z* section of ∼1.50 µm. The images were arranged and labeled using Photoshop software (Adobe).

Mice limbs were frozen in OCT compound and cut into 10 µm-thick sections after overnight treatment at 4° C in 30% sucrose solution. Tissue slices were fixed in 4% paraformaldehyde for 10 min at room temperature. The following antibodies were used: α-CD45 (Biolegend), α-IB4 (Sigma), α-CD31 (BD Biosciences). The sections were then incubated with the appropriate fluorescence-conjugated secondary antibodies (Alexa Fluor 647 or 488, ThermoFisher Scientific), and nuclei were counterstained with DAPI (ThermoFisher Scientific). The samples were mounted using fluorescent mounting medium (Dako). Images were captured and analyzed using a Leica SPEII confocal laser-scanning microscope (Leica). Image acquisition was performed maintaining the same laser power, gain, and offset settings. Multiple independent fields (15–20 for every sections; ×20 or ×40 magnification) per tissue section were randomly chosen and analyzed from at least three limbs for each experimental condition. Image quantification was performed using NIH ImageJ and expressed as the fluorescence area. The microvessel area was quantified by measuring the IB4+ area in the total field area.

### Flow cytometry

To evaluate cell volume, EC were detached with trypsin, washed with PBS and immediately analyzed on a Beckman Coulter Cyan ADP. Forward scatter signal for each cell type was obtained in basal condition and after treatment with inhibitors previously described.

### Chemotaxis assay

Chemotaxis assays with human ECs were performed in a Boyden chamber, as previously described^36^. In brief, PVP-free polycarbonate filters (8 μm pore size; Neuroprobe) were coated with 1% gelatin for 2 h at 37° C. A total of 10 ng/ml VEGF-A dissolved in serum-free medium was seeded in the lower compartment of the chamber; cells were serum starved overnight, and then suspended in serum-free medium at a concentration of 2.5 × 10^6^ cells/ml, and 50 μl of the suspension was added to the upper compartment. After 5 h of incubation at 37° C with 5% CO_2_, the upper surface of the filters was scraped with a rubber policeman, and the filters were fixed and stained with Diff-Quick (Dade Behring). Four random fields of each sample in the lower surface of the filters were counted at ×10 magnification.

### Western blot analysis

For lysates, cells were serum deprived for 2 h and stimulated or not with 30 ng/ml VEGF-A for 15 min. When used, inhibitors were added to cells for 1 h prior to growth factor stimulation. Total proteins were extracted in Laemmli buffer (2.5 mM Tris-HCl, pH 6.8, 2% SDS, 10% glycerol), quantified and equal amounts of each sample were resolved by SDS-PAGE and transferred to PVDF membrane. After blocking with TBS/0.1% Tween 20/5% BSA, membranes were incubated with primary antibody overnight at 4° C. The following primary antibodies were used: rabbit α-pY1175 VEGFR2, α-VEGFR2, α-pS473 Akt, α-pT308 Akt, α-Akt, α-pS235/236 S6, α-pS65 and pT37/46 4EBP1 (all from Cell Signaling Technology), α-β actin (Santa Cruz Biotecnology), α-p110 α (ThermoFisher Scientific). Immunoreactive proteins were identified with secondary antibody coupled to HRP antibody and visualized by ECL. Quantification of the bands was done by NIH ImageJ on representative western blots; band intensities of phosphorylated Akt were normalized on total Akt, and analogously those for phosphorylated VEGFR2 were normalized on total VEGFR2, whereas band intensities of phosphorylated S6 and 4EBP1 were normalized on β actin.

### Spheroid sprouting assay

Spheroid sprouting assay was performed as previously described^37^. In brief, EC spheroids were suspended in medium with or without 20 ng/ml of VEGF-A, and mixed with an equal volume of diluted collagen solution (0.6 mg/ml). Capillary-like sprouts were examined with inverted-phase contrast microscope (Leica) and photographed. To quantify spheroids growth (volume) and sprouting we used the software Ilastik^38^. The software was manually trained on a small dataset of images of spheroids and segmented binary images were obtained. Equivalent radii of the spheroids were measured with a custom written Matlab algorithm, and normalized with control average radius (unstimulated or VEGF-A vehicle-treated, depending on the experiment). To quantify the sprouting, an aspect ratio measure was used, defined as the ratio between the equivalent ratios obtained by perimeter and area of the spheroids.

### Statistical analysis

When not differently indicated, data are presented as average ± standard error (s.e.m.) of three independent experiments. For *in vitro* assays, each experiment was performed with a mix of EC from at least three different umbilical cords. For *in vivo* experiments, cohorts of at least two mice were subjected to each treatment (vehicle/Tamoxifen or vehicle/4-OH Tamoxifen/4-OH Tamoxifen + BEZ235 or Everolimus) and at least three independent experiments were performed. Statistical significance was determined by a Student’s *t*-test.

## Electronic supplementary material

Supplemental figure 1

Supplemental figure 2

Supplemental figure 3

Empty vector EC movie

PIK3CA-WT EC movie

PIK3CA-E545K EC movie

PIK3CA-H1047R EC movie

Supplementary Figure Legends
