# Supplementary figures and images for: PI3K/mTOR inhibition promotes the regression of experimental vascular malformations driven by PIK3CA-activating mutations
Source: Cell Death Dis. 2018 Jan 19;9(2):45. doi: 10.1038/s41419-017-0064-x (PMC5833448; doi:10.1038/s41419-017-0064-x)

A

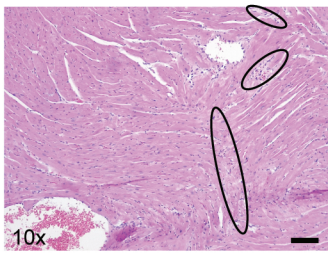

A'

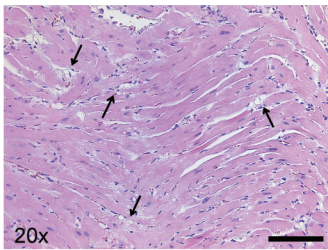

wt/PIK3CA-H1047R  
Cdh5-CRE ERT2 +/-

B

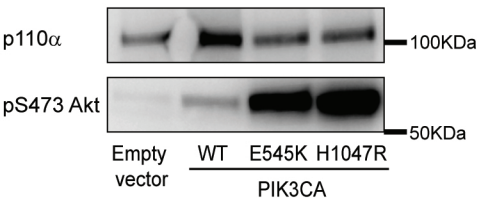

C

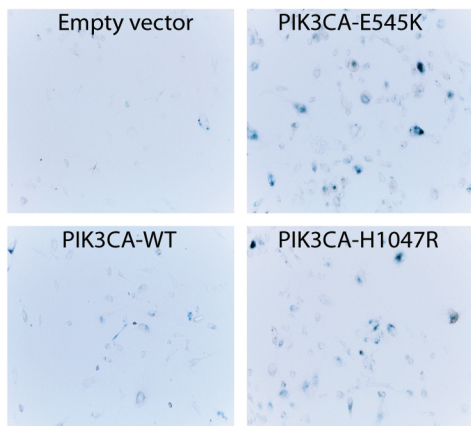

D

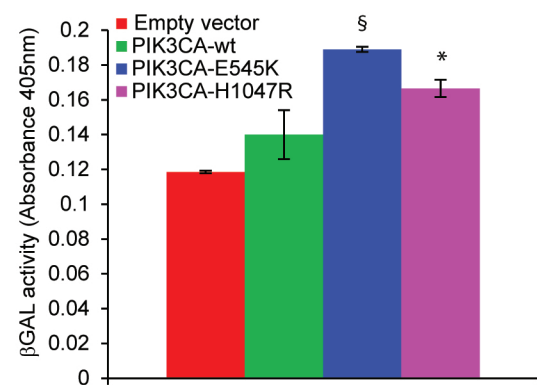

E

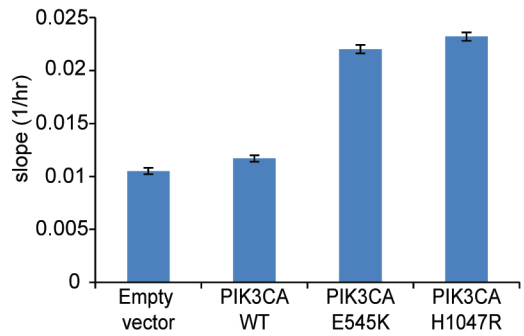

F

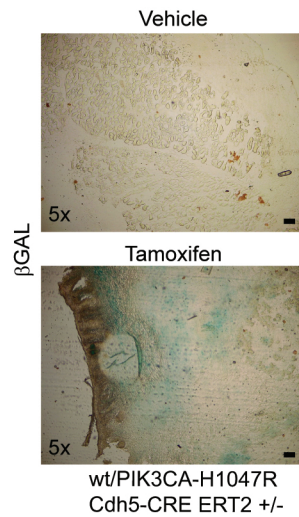

G

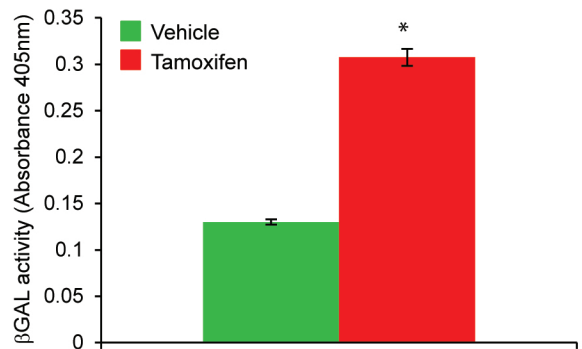

H

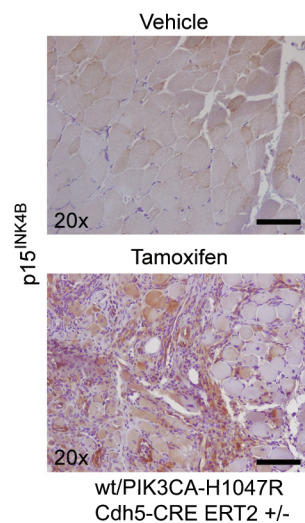

Supplement: Supplementary file 1 — Supplemental figure 1 [file 41419_2017_64_MOESM1_ESM.pdf]

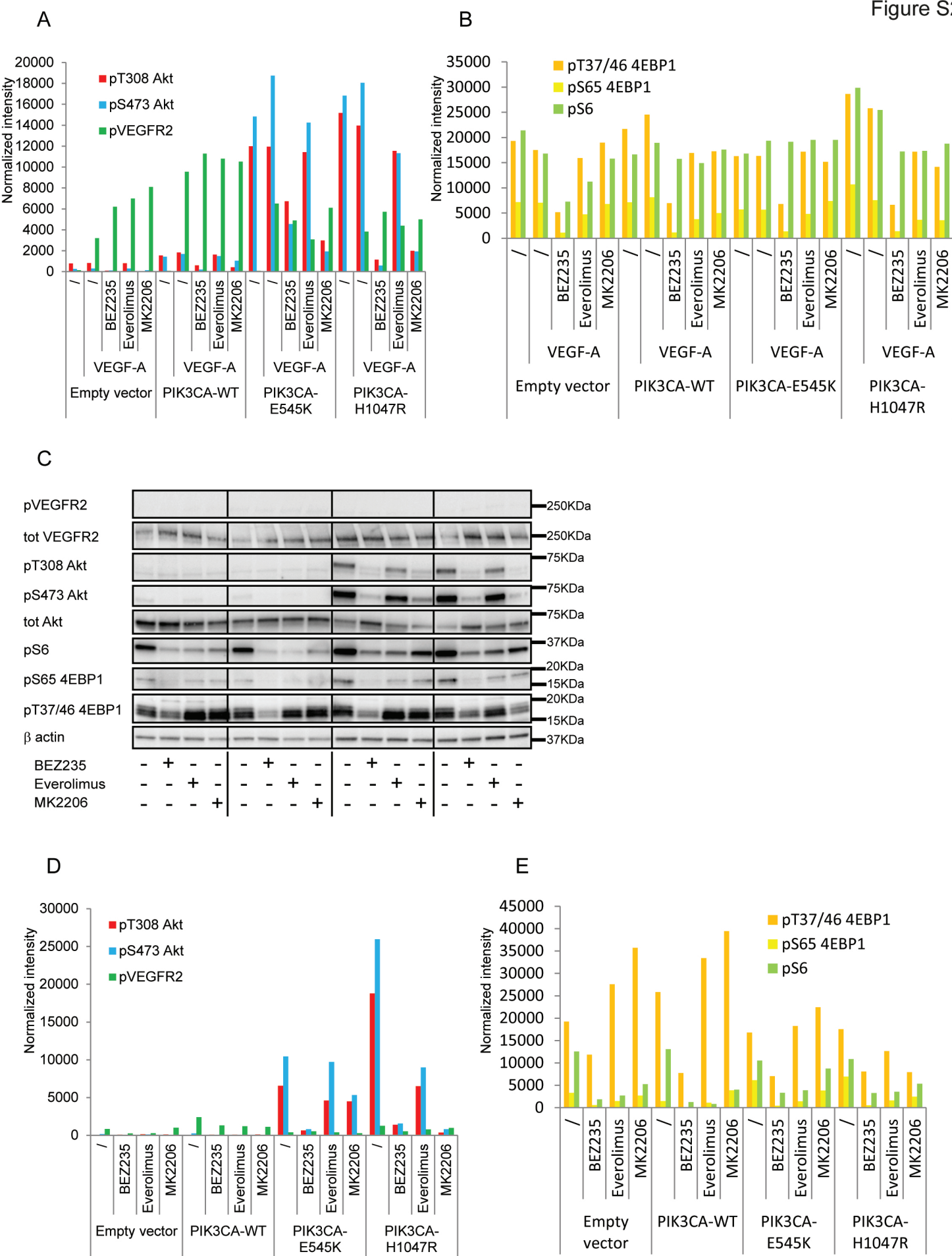

Supplement: Supplementary file 2 — Supplemental figure 2 [file 41419_2017_64_MOESM2_ESM.pdf]

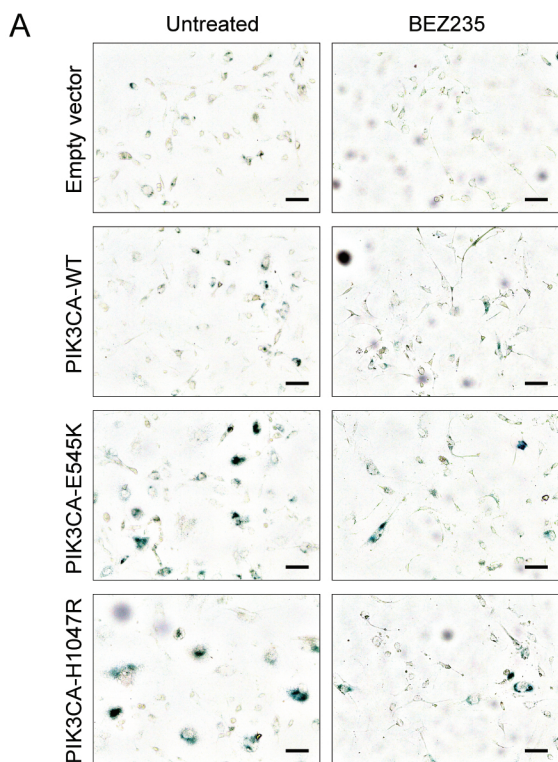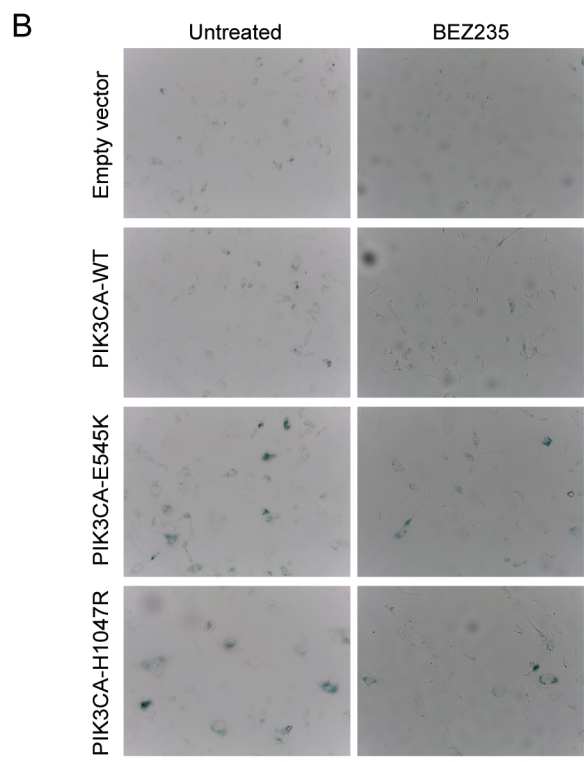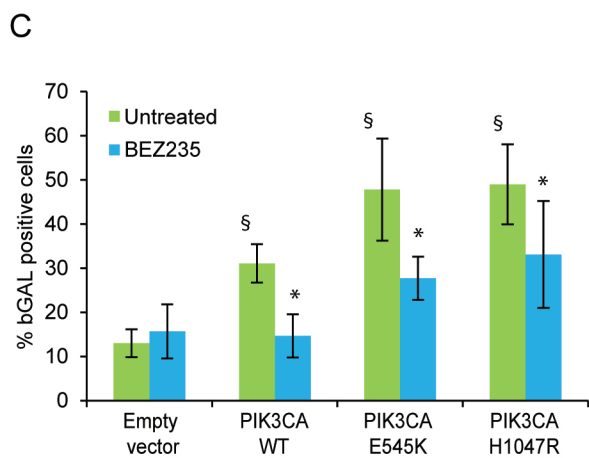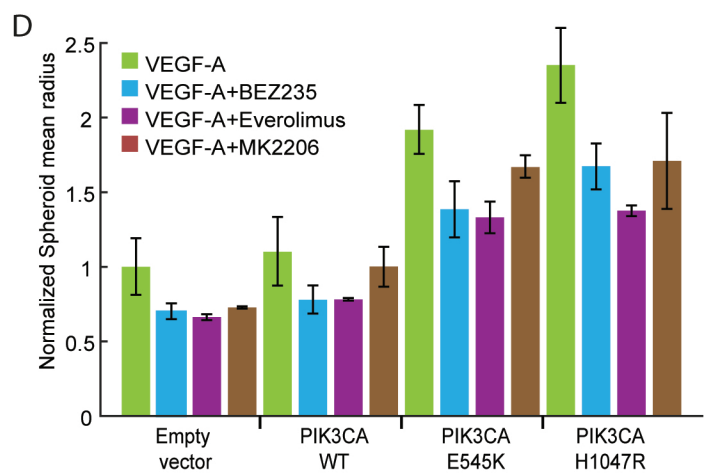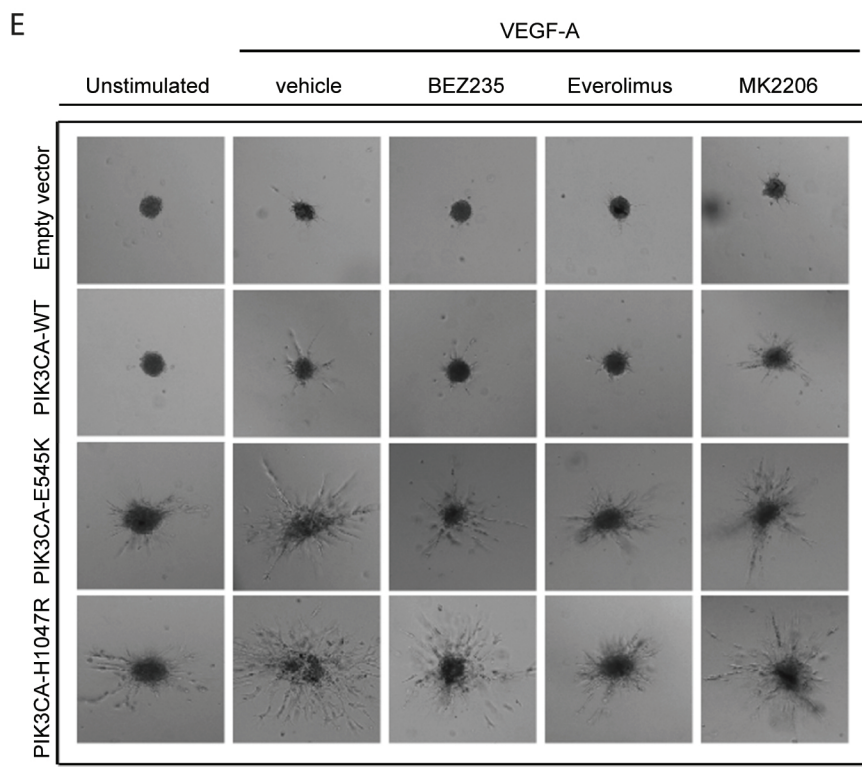

Supplement: Supplementary file 3 — Supplemental figure 3 [file 41419_2017_64_MOESM3_ESM.pdf]
