# Supplementary material for: PI3K/mTOR inhibition promotes the regression of experimental vascular malformations driven by PIK3CA-activating mutations
Source: Cell Death Dis. 2018 Jan 19;9(2):45. doi: 10.1038/s41419-017-0064-x (PMC5833448; doi:10.1038/s41419-017-0064-x)
Supplement: Supplementary file 8 — Supplementary Figure Legends [file 41419_2017_64_MOESM8_ESM.docx]

PI3K/mTOR inhibition promotes the regression of experimental vascular malformations driven by *PIK3CA*-activating mutations

**Supplemental materials**

**Figure S1**. A, A’) *Pik3ca^H1047R^*/ *Cdh5-CreERT2* mice treated with single administration of Tamoxifen were sacrificed after 13 days and heart were dissected and analyzed by H&E staining. B) Lysates of EC infected with retroviruses carrying WT, H1047R or E545K PIK3CA or empty vector were separated by SDS-PAGE and analyzed with the indicated antibodies. C) Original images corresponding to figure 2D. D) Measurement of β-galactosidase activity of EC infected with retroviruses carrying WT, H1047R or E545K PIK3CA or empty vector; §P<0.005 and *P<0.05 versus empty vector EC. E) Growth curve slopes for the indicated cells stimulated with VEGF-A. The slope was calculated as the mean slope between t^0^ and t^m^, where t^0^ is the 18 hours after starting the experiments and t^m^ is 36 hours. F, G) Vehicle or 4-OH Tamoxifen were locally injected in posterior limbs of *Pik3ca*^H1047R^/ *Cdh5-CreERT2* mice. After one week, animals were sacrificed and muscles were dissected and analyzed for β-galactosidase staining (F) and activity (G, *P<0.05 versus vehicle treated). H) Tissues of the same mice were analyzed by immunohistochemistry with anti- p15^INK4B^ antibody.

**Figure S2.** A, B) Quantification of western blots presented in figure 5A. C, D, E) EC were serum starved and then treated with the indicated inhibitors (BEZ235, Everolimus, MK2206); corresponding lysates were then separated by SDS-PAGE and analyzed with the indicated antibodies. Bands were quantified and normalized intensities were plotted.

**Figure S3.** A) β-galactosidase staining showing senescent cells with either no treatment or treated with BEZ235. Image manipulation has been performed to highlight positive cells, as described in the Methods section. B) Original images corresponding to panel A. C) Quantification of the percentage of β-galactosidase -positive cells of experiment shown in panels A-B. Data were plotted as the mean from two independent experiments; §P<0.005 versus untreated empty vector and *P<0.05 versus corresponding untreated cells. D, E) Spheroids of the indicated EC (E) were embedded in a collagen gel and stimulated or not with VEGF-A to generate capillary-like sprouts. VEGF-A stimulated spheroids were also treated with indicate drugs. In D, quantification of spheroids growth and sprouting is shown. Equivalent radii of the spheroids were normalized with control average radius (empty vector EC VEGF-A-stimulated and vehicle-treated).

**Supplementary Movie 1-4.** Time-lapse experiments showing human EC stimulated with VEGF-A without (left) and with (right) BEZ235 treatment. Movie pb: Empty vector; movie wt: PIK3CA WT; movie e: PIK3CA-E545K; movie h: PIK3CA-H1047R.
